# Three-Times-Weekly Administration of Teriparatide Improves Vertebral and Peripheral Bone Density, Microarchitecture, and Mechanical Properties Without Accelerating Bone Resorption in Ovariectomized Rats

**DOI:** 10.1007/s00223-015-9998-0

**Published:** 2015-04-25

**Authors:** Ryoko Takao-Kawabata, Yukihiro Isogai, Aya Takakura, Yukari Shimazu, Emika Sugimoto, Osamu Nakazono, Ichiro Ikegaki, Hiroshi Kuriyama, Shinya Tanaka, Hiromi Oda, Toshinori Ishizuya

**Affiliations:** Laboratory for Pharmacology, Pharmaceuticals Research Center, Asahi Kasei Pharma Corporation, 632-1 Mifuku, Izunokuni, Shizuoka, 410-2321 Japan; Department of Orthopaedic Surgery, Saitama Medical University, 38 Morohongo, Moroyama-cho, Iruma-gun, Saitama, 350-0495 Japan

**Keywords:** Teriparatide, Three-times-weekly, Bone metabolism, Mechanical strength, Micro-computed tomography

## Abstract

Daily and weekly administration of teriparatide (PTH_1–34_) reduces the risk of osteoporotic bone fractures. However, their effects on markers of bone formation and bone resorption differ. These results indicate that the dosing frequency of teriparatide may affect bone metabolism and bone structure, with different effects on bone strength. In the present study, to evaluate the dose-related effects of a low administration frequency of teriparatide on bone status, we investigated the effects of three-times-weekly administration of teriparatide (1.1, 5.6, or 28.2 µg/kg) for 12 months on bone parameters, including bone metabolism markers, bone mineral density (BMD), micro-computed tomography, and bone strength, using 6-month-old ovariectomized (OVX) rats. Three-times-weekly administration of teriparatide dose-dependently increased the BMD of the lumbar vertebra and femur in OVX rats, and increased serum osteocalcin (a marker of bone formation), but not type I collagen C-telopeptide (a marker of bone resorption). The trabecular number and thickness increased in the vertebrae and femur, as in prior reports of daily teriparatide administration in OVX rats. Cortical thickness increased only toward the endocortical side of the femur, unlike with daily administration. Bone strength of the vertebrae and proximal and shaft of the femur was correlated with the changes in BMD and bone structure. These results demonstrate the effects of low frequency, intermittent administration of teriparatide on the biomechanical, and microstructural properties of bone in OVX rats.

**Electronic supplementary material:**

The online version of this article (doi:10.1007/s00223-015-9998-0) contains supplementary material, which is available to authorized users.

## Introduction

Osteoporosis is a skeletal disorder characterized by compromised bone strength and increased risk of fracture, which impair daily activities and quality of life [1]. In the 2000s, daily injections of teriparatide (parathyroid hormone; PTH_1–34_) [2] and full-length PTH (PTH_1–84_) [3] were approved for the treatment of osteoporosis. More recently, in 2011, once-weekly administration of teriparatide was approved in Japan, and demonstrated marked reductions in the risk of vertebral fracture that are comparable to daily administration.

PTH exerts anabolic effects on bone by promoting the proliferation and differentiation of osteoblasts, and decreasing apoptosis. However, PTH also indirectly promotes bone resorption by inducing the differentiation of osteoclasts. The balance between the promotion of bone formation and bone resorption is defined by the blood PTH concentration profile, because bone resorption is preferentially promoted when the blood PTH concentration is persistently high, and bone formation is preferentially promoted when the PTH concentrations are intermittently high, leading to anabolic effects on bone. Furthermore, it has been suggested that the effects of PTH on the bone differ according to the dosing regimen of intermittent administration [1, 4, 5].

In osteoporosis patients, daily treatment with teriparatide and PTH_1–84_ elicits greater increases in bone formation markers (≥80 % for serum bone-specific alkaline phosphatase concentration relative to the pre-treatment levels) than in bone resorption markers (≥100 % for urinary type I collagen N-telopeptide (NTX/creatinine) relative to the pre-treatment level), thereby accelerating bone turnover, and promoting an increase in bone mineral density (BMD) [3, 6]. However, once-weekly administration of teriparatide was reported to increase bone formation markers to a lesser degree (by about 20 % for serum osteocalcin and 15 % for serum procollagen type I N propeptide relative to the placebo group) when compared with daily administration of teriparatide [7]. Unexpectedly, once-weekly administration decreased bone resorption markers compared with placebo [7]. These results indicate that the modest bone turnover associated with once-weekly administration of teriparatide could change the bone microstructure, and may improve bone strength via a different mechanism compared with daily administration. However, the effects of teriparatide with a lower administration frequency on bone architecture and mechanical strength are unclear. To our knowledge, no studies have examined the long-term (>6 months) effects of PTH administration in ovariectomized (OVX) rats with a lower administration frequency, such as three-times-weekly administration, where the dosing intervals are >2 days. Therefore, to understand the long-term effects of a lower frequency of teriparatide administration on bone status, we conducted the present study in which rats were treated with teriparatide three-times-weekly for 12 months. We assessed the dose-related effects on the BMD changes in vivo, bone metabolism markers, bone densities of the vertebral body, tibia, and femur by dual-energy X-ray absorptiometry, microstructure of trabecular bone and cortical bone by micro-computed tomography (micro-CT), and bone mechanical strength.

## Materials and Methods

### Animals and Methods

All experiments were approved by the experimental animal ethics committee at Asahi Kasei Pharma Corporation and were conducted in accordance with the guidelines for the management and handling of experimental animals. The animal work described below and the analysis of bones were performed as part of the development program for once-weekly administration of teriparatide.

Eleven-week-old virgin female Sprague–Dawley rats (Crl:CD(SD)) were purchased from Charles River Japan (Atsugi, Japan) and were allowed to acclimatize for 2 weeks before use. Animals were housed individually under a 12-h light/dark cycle with free access to water and food (CRF-1, standard diet for rats; Oriental Yeast, Tokyo, Japan) throughout the study. At 13 weeks of age, sexually mature rats underwent bilateral ovariectomy or sham surgery, as previously reported [8], under pentobarbital anesthesia comprising intraperitoneal injection with approximately 35 mg/kg of pentobarbital. For sham surgery, approximately 10-mm-long incisions were made on the back of the rats, as in ovariectomy, and the ovaries were exposed and then replaced. The rats were carefully observed for 10 days after the surgery. There were no significant abnormalities in the general conditions or food consumption following surgery. Post-operative analgesia was not used.

The rats were divided into eight groups: a start-control group (*n* = 10) of 3-month-old (−3 M) rats, two sham groups, and five OVX groups. The sham and OVX animals were left untreated for 3 months to establish osteoporosis. One sham group and one OVX group (baseline-S group and baseline-O group, *n* = 10 per group) were killed at 6 months of age (0 M) as baseline controls to confirm the onset of osteoporosis. The remaining five groups received three-times-weekly subcutaneous injections for 12 months: One sham group and one OVX group (sham group and OVX group, *n* = 15 per group) were treated with saline as a vehicle. The remaining three OVX groups were treated with 1.1 μg/kg (low-dose teriparatide group), 5.6 μg/kg (medium-dose teriparatide group), or 28.2 μg/kg (high-dose teriparatide group) of teriparatide (Asahi Kasei Pharma Corporation, Tokyo, Japan) prepared in saline. The 5.6 μg/kg dose of teriparatide is equivalent to a 6 μg/kg dose of teriparatide acetate, which prevented ovariectomy-induced reductions in femoral ash weight when administered three-times-weekly to OVX rats for 25 weeks [9]. The other two doses were included to provide a dose response.

At the end of each experimental period (−3, 0, and 12 M), the animals scheduled for euthanasia at the specific time underwent terminal blood sampling by abdominal aortic puncture under anesthesia. At 12 M, blood samples were obtained 2 days after the last dose. Serum samples were stored at −80 °C until used to measure biochemical markers (Electronic Supplementary Materials). The fourth lumbar vertebra (L4) and right femur were collected and stored at −30 °C.

All animals were examined by pathologists at the necropsy, and any tissues and organs with macroscopic abnormalities were examined histopathologically. The cause of death of any animal that died during the study was determined wherever possible.

### Bone Mineral Density (BMD) Changes in the Tibia

The BMD of the tibia was measured before surgery (−3 M), and at 0, 3, 6, and 12 months by dual-energy X-ray absorptiometry (DXA; DCS-600EX-3R, Aloka, Tokyo, Japan) under ketamine and xylazine anesthesia (approximately 6.7 mg/kg ketamine and 3.3 mg/kg xylazine, intramuscular). The rats were placed in the prone position on the DXA scanning table. The left tibia was scanned at a pitch of 1 mm and scan speed of 25 mm/min. BMD was calculated from the values for bone mineral content and bone area for three regions of the tibia (proximal, shaft, and distal). Further details are presented in the Electronic Supplementary Materials.

### Preparation of Bone Samples

The isolated bone samples (femur and L4) were soaked at room temperature and cleaned to remove adherent soft tissue. For one femoral specimen in the sham group, the epiphyseal region was damaged during the cleaning, and we excluded this specimen from the analysis. The L4 was resected at the vertebral arch, and the transverse and spinous processes. A central cylinder specimen with planoparallel ends and a height of 3.7 ± 0.1 mm was obtained from each vertebral body using a diamond band saw (BS-3000; Exakt, Norderstedt, Germany). Five lumbar specimens (two in the start-control group, two in the baseline-S group, and one in the baseline-O group) could not be trimmed properly, and we could not obtain cylinders from these samples. The femur length was measured using digital calipers and femoral volume was determined using Archimedes’ principle. BMD, micro-CT, bone mechanical properties, and finite elemental analysis (FEA) of the isolated bone samples were assessed as described below. FEA and correlation analysis were performed after once-weekly teriparatide was approved for clinical use.

### Micro-CT

A cone-beam X-ray micro-CT system (ScanXmate-RB090SS150; Comscantecno, Kanagawa, Japan) was used to obtain CT images of the isolated bone samples using the following settings: tube voltage, 70 kV; tube current, 0.1 mA; and voxel size, 11.8 µm^3^ (lumbar vertebra and femoral shaft) or 19.3 µm^3^ (proximal femur). Three-dimensional images were reconstructed and analyzed using TRI/3D-BON software (RATOC System Engineering, Tokyo, Japan). For the lumbar vertebra, we analyzed the middle portion (height, 2 mm) of the lumbar vertebral cylinder. For the femoral neck, we analyzed the central portion of the neck region (height, 0.3 mm) perpendicular to the axis, as previously reported [10]. For the femoral intra-trochanter, we analyzed a region (height, 0.3 mm) 1.0 mm distal from the deepest part of the trochanteric fossa, perpendicular to the axis of the femoral shaft. For the femoral shaft, we analyzed a region (height, 1.0 mm) on the plane perpendicular to the shaft at a point halfway along the length of the femur.

The trabecular bone structure was determined in the lumbar vertebra, femoral neck, and intra-trochanter, and the following parameters were measured: cancellous bone volume (BV/TV, %), trabecular thickness (Tb.Th, μm), trabecular number (Tb.N, 1/mm), trabecular bone pattern factor (TBPf, 1/mm) [11], and structure model index (SMI) [12]. The cortical bone structure was determined in the femoral neck, intra-trochanter, and femoral shaft, and the following parameters were measured: cortical bone ratio (Cv/Av, %), cortical bone thickness (Ct.Th, μm), external length (Ex.L, μm), internal length (In.L, μm), cortical void volume (Vv/Cv, %), and second moment of inertia (moment, mm^5^).

### Bone Mechanical Properties

Compression testing of the vertebral body was performed as previously described [13]. Briefly, the lumbar vertebral cylinder specimens were placed on a lower platen with the cranial side facing up, and were compressed with an upper platen, 4 mm in diameter, using a material testing machine (EZ-L-1kN; Shimadzu, Tokyo, Japan) at a constant speed of 2 mm/min. The load and displacement curve were recorded and the following parameters were calculated using TRAPEZIUM2 software (Shimadzu): maximum load (N), stiffness (N/mm), and breaking energy (Energy, N·mm).

The femoral shaft was subjected to a three-point bending test, as previously described [14], using the material testing machine at a constant speed of 10 mm/min. The load and displacement curve were recorded and the following parameters were calculated: maximum load (N), stiffness (N/mm), and breaking energy (Energy, N·mm).

After the three-point bending test, mechanical testing of the femoral neck was performed using the material testing machine, as previously described [15]. The shaft of the proximal femur was vertically embedded in dental resin. The specimen was placed on a lower platen with the proximal part facing up and compressed with an upper platen, 4 mm in diameter, at a constant speed of 2 mm/min. The load and displacement curve were recorded and the following parameters were calculated: maximum load (N), stiffness (N/mm), and breaking energy (Energy, N·mm).

### Finite Element Analysis (FEA)

The reconstructed three-dimensional grayscale images of the proximal femur obtained by micro-CT (550 slices with a voxel size of 19 µm) were used for FEA. A compressive force of 100 N was applied by a rod (1 mm in diameter) axially to the top of the femoral head in the model. Before conducting FEA, the fracture stress was validated based on the mechanical properties of the femoral specimens of sham and OVX rats.

In fracture load analysis, von Mises stress >144 MPa was defined as the stress that induces bone fracture. Using TRI/3D-FEM64 FEA software (RATOC System Engineering) [16, 17], the fracture load was determined as the level that induces fractures in 2.8 % of all voxels. The Young’s modulus for each element was assumed to be isotropic and we used a cubic BMD function, varying from 0 GPa for no bone, 3.57 GPa for a BMD of 600 mg/cm^3^, and 22 GPa for a BMD of 1100 mg/cm^3^, where Young’s modulus (GPa) = 16.53 (BMD)^3^ [16]. A Poisson’s ratio of 0.3 was incorporated in the model.

### Statistical Analysis

All data are presented as means ± standard deviation (SD). The sham and OVX groups were compared using the unpaired Student’s *t* test, and *P* values <0.05 were considered significant. Based on a previous report [9] and our preliminary studies, BMD and bone mechanical properties were assumed to increase with three-times-weekly administration of teriparatide in a dose-dependent manner. For these parameters, the teriparatide-treated groups were compared with the OVX groups using the Williams’ test, a one-sided statistical method used to compare multiple dose groups with a control group [18], and *P* values <0.025 were considered statistically significant. For BMD changes in tibia, the Williams’ test was performed at each time point following the confirmation of significance in one-way analysis of variance (ANOVA). For other variables, the teriparatide-treated groups were compared with the OVX groups using the Dunnett’s test, and *P* values of <0.05 were considered statistically significant. The relationship between mechanical properties (maximum load) and BMD or structural parameters were analyzed for the lumbar vertebra, proximal femur, and femoral shaft for all OVX rats combined (*n* = 58) using simple linear regression. All analyses were performed using SAS, Version 8.2 (SAS Institute Inc., Cary, NC, USA).

## Results

### Treatment Period and Outcomes of Ovariectomy

There were no clinical signs of treatment-related toxicity during the treatment period. Three rats in the sham group and two rats in the high-dose teriparatide group died during the treatment period. The causes of the death were anterior pituitary tumor (two rats in the sham group and one rat in the high-dose teriparatide group) and leukemia (large granular cell leukemia in one rat in the sham group; and one rat in the high-dose teriparatide group had leukemia, which could not be classified due to the marked postmortem changes). These tumors occur spontaneously in this strain and were deemed unrelated to the treatment. No skeletal abnormalities, such as osteosarcoma, were observed during the study. The rats in the OVX groups weighed approximately 20 % more than those in the sham group at the end of the study. Teriparatide did not affect body weight. At the end of the study, the OVX rats had uterine atrophy. Neither ovariectomy nor teriparatide affected the length or volume of the femur. The mean femoral lengths were 34.8, 37.0, and 37.2 mm in the start-control, baseline-S, and baseline-O groups, respectively. At the end of the study, the mean femoral length ranged from 37.8 to 38.6 mm in the sham, OVX, and teriparatide groups.

### BMD Changes in the Tibia

**Fig. 1:**
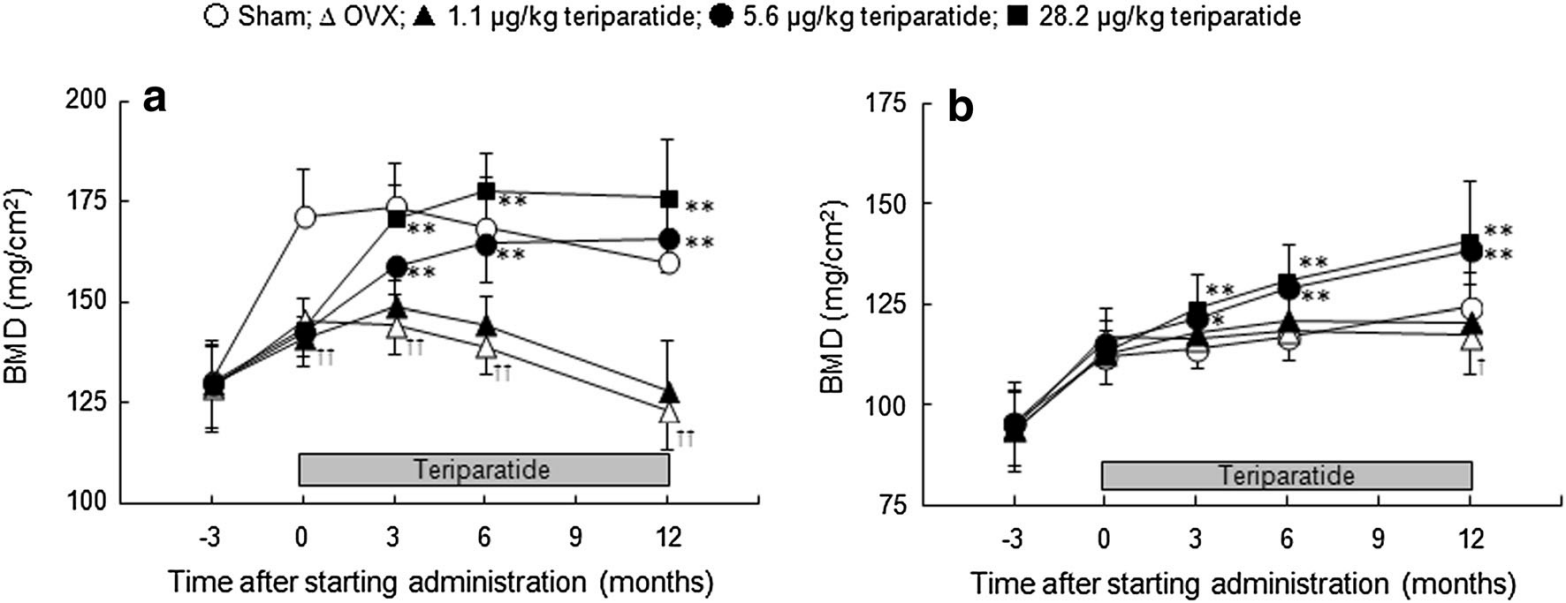
BMD changes in the proximal tibia (**a**) and the tibial shaft (**b**). *P < 0.05 and **P < 0.01: teriparatide versus OVX (Williams’ test following one-way ANOVA); ^†^ *P* < 0.05 and ^††^ *P* < 0.01: OVX versus sham (*t* test)

The BMD of the proximal tibia, which is rich in trabecular bone, was consistently and significantly lower in the OVX group than in the sham group (*P* < 0.01) from 3 months after ovariectomy (i.e., 0 M) to the end of treatment (12 M) (Fig. 1a). The BMD of the tibial shaft, which is rich in cortical bone, was lower at 15 months after ovariectomy (i.e., 12 M) but not at earlier times (Fig. 1b). Administration of teriparatide increased the BMD of the proximal and shaft of the tibia in a dose-dependent manner (Fig. 1a, b). The BMD of the proximal tibia in the medium- and high-dose teriparatide groups reached the level of the sham group after 6 and 3 months of treatment, respectively, and continued to increase throughout the study. The BMD of the tibial shaft in the medium- and high-dose teriparatide groups exceeded the level in the sham group after 3 months of treatment, and continued to increase throughout the study.

### Serum Biochemistry

**Fig. 2:**
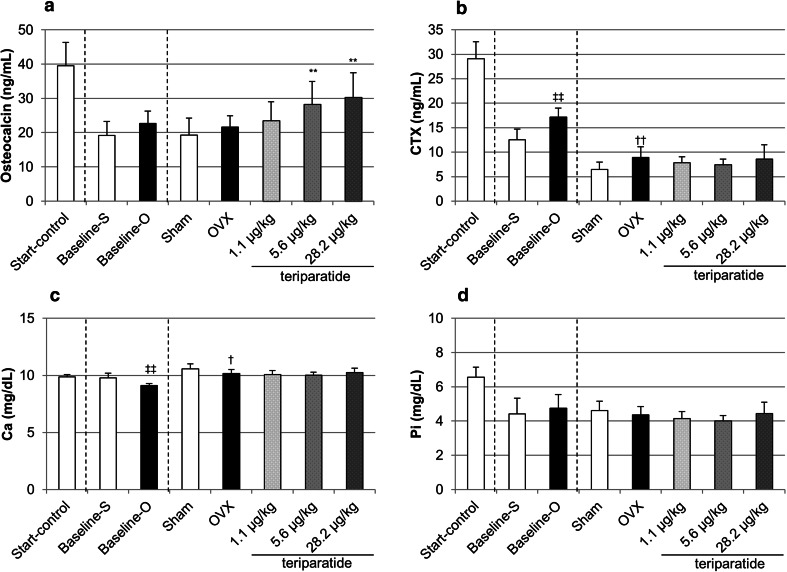
Serum concentrations of osteocalcin (**a**), CTX (**b**), Ca (**c**), and P_i_ (**d**) at the end of the treatment period. **P < 0.01: teriparatide versus OVX (Dunnett’s test); ^†^ *P* < 0.05 and ^††^ *P* < 0.01: OVX versus sham (*t* test); ^‡‡^ *P* < 0.01: baseline-O versus baseline-S (*t* test)

Osteocalcin levels tended to be higher in the baseline-O and OVX groups than in the baseline-S and sham groups, respectively, but the differences were not significant. Teriparatide increased osteocalcin in a dose-dependent manner, with significant increases observed in the medium- and high-dose teriparatide groups (Fig. 2a). Serum type I collagen C-telopeptide (CTX) levels were significantly higher in the baseline-O and OVX groups than in the baseline-S and sham groups, indicating that accelerated bone resorption continued for 15 months after ovariectomy. There were no significant differences in CTX between the teriparatide-treated and OVX groups (Fig. 2b). Serum calcium (Ca) levels were slightly but significantly lower in the baseline-O and OVX groups than in the baseline-S and sham groups. The serum Ca levels were not significantly different between the teriparatide-treated and OVX groups (Fig. 2c). Serum inorganic phosphorous (P_i_) were not significantly different among the study groups (Fig. 2d).

### Effects of Ovariectomy on BMD and Micro-CT

**Fig. 3:**
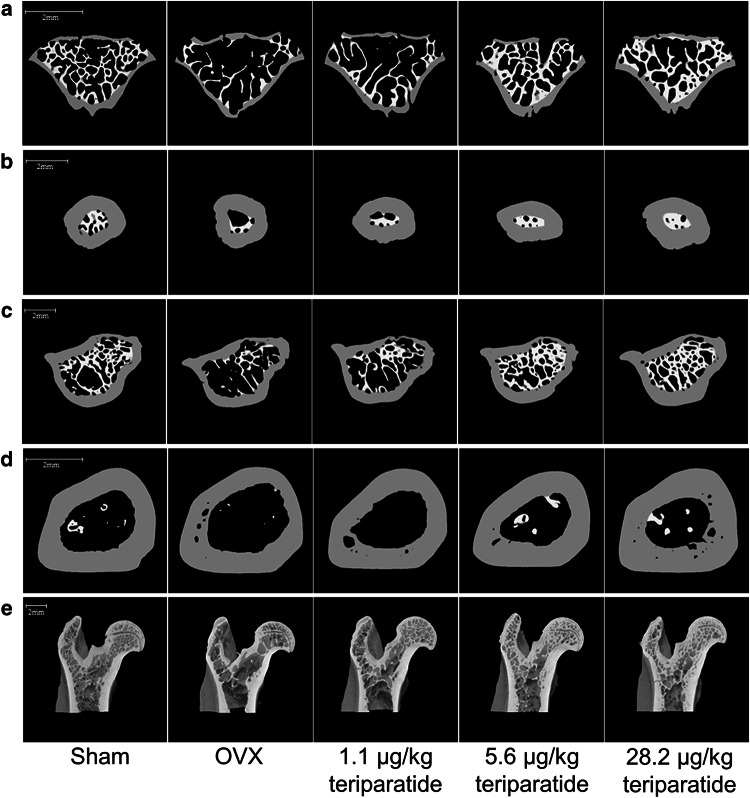
Cross-sectional micro-CT images of the lumbar vertebral body (**a**), femoral neck (**b**), inter-trochanter (**c**), femoral shaft (**d**), and vertical image of the proximal femur (**e**). Cross-sectional bone images are *white* for trabecular bone and *gray* for cortical bone. *Scale bar* 2 mm

As expected, ovariectomy-induced osteoporosis according to the changes in BMD and micro-CT (Tables 1–3; Fig. 3; Electronic Supplementary Materials).

**Table 1:** Characteristics of the lumbar vertebra

	Sham	OVX	1.1 μg/kg teriparatide	5.6 μg/kg teriparatide	28.2 μg/kg teriparatide
*n*	12	15	15	15	13
DXA					
BMC (mg)	17.1 ± 2.6	10.6 ± 1.4^††^	12.3 ± 1.5*	17.4 ± 1.9**	18.9 ± 2.5**
BMD (mg/cm^2^)	63.1 ± 7.5	44.2 ± 3.9^††^	49.0 ± 3.8*	62.7 ± 6.0**	68.8 ± 7.1**
Micro-CT					
Trabecular bone					
BV/TV (%)	35.4 ± 4.4	16.2 ± 3.3^††^	22.1 ± 2.7*	34.1 ± 6.6**	41.6 ± 8.2**
Tb.Th (μm)	86 ± 6	87 ± 8	92 ± 9	107 ± 7**	110 ± 8**
Tb.N (1/mm)	2.34 ± 0.15	1.27 ± 0.26^††^	1.50 ± 0.15*	1.73 ± 0.27**	1.89 ± 0.26**
TBPf (1/mm)	−1.97 ± 1.87	4.20 ± 2.21^††^	0.61 ± 1.39**	−3.04 ± 1.84**	−5.67 ± 2.38**
SMI	0.50 ± 0.27	1.63 ± 0.43^††^	1.16 ± 0.27*	0.59 ± 0.47**	−0.01 ± 0.66**
Mechanical properties (compressive test)					
Maximum load (N)	336 ± 68	220 ± 38^††^	265 ± 45	419 ± 65**	485 ± 101**
Stiffness (N/mm)	2152 ± 316	1787 ± 275^††^	1887 ± 352	2338 ± 248**	2298 ± 237**
Energy (N·mm)	32.2 ± 9.5	18.0 ± 4.2^††^	24.0 ± 5.1	46.5 ± 11.2**	63.6 ± 20.8**

Values are shown as means ± SD

*Sham* sham surgery, *OVX* ovariectomy, *BMC* bone mineral content, *BMD* bone mineral density, *BV/TV* cancellous bone volume/tissue volume, *Tb.Th* trabecular thickness, *Tb.N* trabecular number, *TBPf* trabecular bone pattern factor, *SMI* structure model index

* *P* < 0.05 and ** *P* < 0.01: teriparatide versus OVX (Williams’ test for bone density and mechanical properties; Dunnett’s test for micro-CT); ^††^ *P* < 0.01: OVX versus sham (*t* test)

### Effects of Teriparatide on BMD and Micro-CT of the Lumbar Vertebra

Teriparatide significantly and dose-dependently increased BMD of the lumbar vertebra groups (Table 1). Micro-CT images of the lumbar vertebra showed that teriparatide increased BV/TV in a dose-dependent manner by increasing both Tb.N and Tb.Th compared with the OVX group. Teriparatide also decreased the TBPf and the SMI in a dose-dependent manner, corresponding to restoration of the plate-like structure of trabecular bone and an increase in trabecular connectivity (Fig. 3a; Table 1).

### Effects of Teriparatide on BMD and Micro-CT of the Proximal Femur

As expected, the BMD of the proximal femur was significantly lower in the OVX group than in the sham group. The reduction in BMD was accompanied by a reduction in the proximal femur’s mechanical strength, indicating that ovariectomy induced osteoporosis [19]. Teriparatide significantly and dose-dependently increased the BMD of the proximal femur compared with the OVX group (Table 2).

**Table 2:** Characteristics of the proximal femur

	Sham	OVX	1.1 μg/kg teriparatide	5.6 μg/kg teriparatide	28.2 μg/kg teriparatide
*n*	12	15	15	15	13
DXA					
BMC (mg)	147 ± 16	124 ± 10^††^	123 ± 7	148 ± 14**	160 ± 15**
BMD (mg/cm^2^)	140 ± 10	119 ± 6^††^	121 ± 5	140 ± 8**	150 ± 8**
Micro-CT					
Femoral neck					
Trabecular bone					
BV/TV (%)	65.3 ± 6.7	43.2 ± 14.7^††^	48.3 ± 10.5	71.6 ± 15.3**	89.6 ± 10.3**
Tb.Th (μm)	129 ± 14	125 ± 20	129 ± 14	158 ± 29**	167 ± 36**
Tb.N (1/mm)	2.01 ± 0.49	1.89 ± 0.98	1.94 ± 0.48	2.54 ± 0.74	3.22 ± 0.96**
TBPf (1/mm)	−9.0 ± 2.1	−2.8 ± 3.2^††^	−5.2 ± 2.2	−10.9 ± 5.0**	−17.8 ± 6.2**
SMI	1.57 ± 0.20	2.310 ± 0.30^††^	2.23 ± 0.25	2.10 ± 0.40	2.24 ± 0.42
Cortical bone					
Cv/Av (%)	78.5 ± 4.4	81.8 ± 7.4	84.5 ± 5.6	86.0 ± 5.5	90.1 ± 3.6**
Ct.Th (μm)	552 ± 37	576 ± 54	597 ± 40	639 ± 57**	703 ± 61**
Ex.L (mm)	6.96 ± 0.39	6.69 ± 0.52	6.58 ± 0.35	6.77 ± 0.32	6.74 ± 0.39
In.L (mm)	3.29 ± 0.49	2.98 ± 0.82	2.73 ± 0.56	2.65 ± 0.61	2.20 ± 0.46**
Vv/Cv (%)	0.2 ± 0.1	0.2 ± 0.4	0.1 ± 0.1	0.1 ± 0.2	0.3 ± 0.7
Femoral intra-trochanter					
Trabecular bone					
BV/TV (%)	43.5 ± 5.8	22.7 ± 6.0^††^	28.3 ± 3.3	43.1 ± 7.0**	50.8 ± 8.3**
Tb.Th (μm)	123 ± 11	114 ± 7^††^	110 ± 8	121 ± 9*	129 ± 9**
Tb.N (1/mm)	1.06 ± 0.16	0.70 ± 0.22^††^	0.78 ± 0.13	0.99 ± 0.18**	1.17 ± 0.24**
TBPf (1/mm)	−4.49 *±* 1.78	1.37 ± 1.50^††^	−1.67 ± 1.10**	−4.33 ± 1.39**	−5.19 ± 1.92**
SMI	1.21 ± 0.21	1.93 ± 0.19^††^	1.55 ± 0.15*	1.25 ± 0.18**	1.2 ± 0.28**
Cortical bone					
Cv/Av (%)	49.4 ± 2.3	44.3 ± 3.0^††^	45.9 ± 2.7	50.6 ± 2.8**	55.8 ± 3.0**
Ct.Th (μm)	650 ± 40	579 ± 50^††^	592 ± 43	680 ± 45**	758 ± 49**
Ex.L (mm)	17.8 ± 0.6	18.0 ± 0.6	17.7 ± 0.4	18.1 ± 0.6	17.9 ± 0.6
In.L (mm)	12.7 ± 0.5	13.4 ± 0.4^††^	12.9 ± 0.4*	12.7 ± 0.5**	11.9 ± 0.5**
Vv/Cv (%)	2.1 ± 0.5	4.5 ± 1.5^††^	3.8 ± 0.6	3.3 ± 1.1*	2.3 ± 0.6**
Mechanical properties					
Maximum load (N)	117 ± 23	106 ± 13^††^	110 ± 15	125 ± 21**	130 ± 24**
Stiffness (N/mm)	514 ± 109	448 ± 59	461 ± 63	521 ± 56**	546 ± 53**
Energy (N·mm)	16.2 ± 3.9	17.0 ± 4.1	16.6 ± 3.5	17.9 ± 5.1	18.2 ± 5.2

Values are shown as means ± SD

*Sham* sham surgery, *OVX* ovariectomy, *BMC* bone mineral content, *BMD* bone mineral density, *BV/TV* cancellous bone volume/tissue volume, *Tb.Th* trabecular thickness, *Tb.N* trabecular number, *TBPf* trabecular bone pattern factor, *SMI* structure model index, *Cv/Av* cortical bone ratio, *Ct.Th* cortical bone thickness, *Ex.L* external length, *In.L* internal length, *Vv/Cv* cortical void volume

* P < 0.05 and ** *P* < 0.01: teriparatide versus OVX (Williams’ test for bone density and mechanical properties; Dunnett’s test for micro-CT); ^††^ *P* < 0.01: OVX versus sham (*t* test)

Micro-CT images of the proximal femur are shown in Fig. 3b, c, and e. Structural analysis of the femoral neck revealed that BV/TV, Tb.Th, and Tb.N were higher and TBPf was lower in the teriparatide groups than in the OVX group, suggesting that teriparatide induced favorable changes in the structure and connectivity of trabecular bone. SMI was not significantly different between the teriparatide-treated and OVX groups. Changes in BV/TV, Tb.Th, and TBPf were observed at doses ≥5.6 μg/kg, whereas the change in Tb.N was only observed at the dose of 28.2 μg/kg (Table 2).

The Ex.L and Vv/Cv were not affected by teriparatide. However, teriparatide improved Ct.Th at doses ≥5.6 μg/kg, and Ct/Av and In.L were improved at a dose of 28.2 μg/kg (Table 2) compared with the OVX group. Teriparatide also increased BV/TV, Tb.Th, and Tb.N, and decreased TBPf and SMI in the femoral intra-trochanter compared with the OVX group (Table 2), suggesting that teriparatide improved the structure of trabecular bone. The changes in TBPf and SMI occurred at doses ≥1.1 μg/kg, and the changes in BV/TV, Tb.Th, and Tb.N occurred at doses ≥5.6 μg/kg (Table 2). Additionally, teriparatide increased Cv/Av and Ct.Th, and decreased In.L and Vv/Cv, but did not affect Ex.L of the intra-trochanter cortical bone compared with the OVX group. These differences were observed at doses ≥1.1 μg/kg for In.L, and at doses ≥5.6 μg/kg for Cv/Av, Ct.Th, and Vv/Cv (Table 2). These findings indicate that teriparatide increased intra-trochanter endocortical bone formation, increased cortical bone thickness and volume, and decreased cortical porosity.

### Effects of Teriparatide on BMD and Micro-CT of the Femoral Shaft

As shown in Table 3, teriparatide increased the BMD of the femoral shaft in a dose-dependent manner compared with the OVX group. Differences between the teriparatide-treated and OVX groups were apparent with doses ≥1.1 μg/kg (Table 3). Micro-CT images of the femoral shaft are shown in Fig. 3d. Teriparatide significantly and dose-dependently increased Cv/Av and Ct.Th, and decreased In.L compared with the OVX group, but did not affect Ex.L, Vv/Cv, or Moment (Table 3).

**Table 3:** Characteristics of the femoral shaft

	Sham	OVX	1.1 μg/kg teriparatide	5.6 μg/kg teriparatide	28.2 μg/kg teriparatide
*n*	11	15	15	15	13
DXA					
BMC (mg)	129 ± 18	115 ± 11^†^	118 ± 7	137 ± 13**	148 ± 11**
BMD (mg/cm^2^)	149 ± 14	132 ± 9^††^	138 ± 5*	156 ± 10**	168 ± 7**
Micro-CT					
Cortical bone					
Cv/Av (%)	67.4 ± 3.5	56.7 ± 5.4^††^	63.9 ± 3.1**	70.4 ± 1.9**	75.9 ± 2.7**
Ct.Th (μm)	864 ± 77	703 ± 85^††^	806 ± 56**	937 ± 60**	1039 ± 59**
Ex.L (mm)	13.6 ± 0.7	14.0 ± 0.6	13.6 ± 0.4	13.8 ± 0.6	13.7 ± 0.4
In.L (mm)	8.0 ± 0.6	9.5 ± 0.8^††^	8.3 ± 0.5**	7.7 ± 0.5**	7.0 ± 0.6**
Vv/Cv (%)	0.4 ± 0.6	2.0 ± 2.4	3.0 ± 2.5	1.4 ± 1.6	1.0 ± 1.1
Moment (mm^5^)	12.8 ± 2.8	12.7 ± 1.7	12.1 ± 1.5	13.8 ± 2.1	13.6 ± 1.8
Mechanical properties					
Maximum load (N)	246 ± 46	192 ± 40^††^	226 ± 41*	263 ± 33**	292 ± 47**
Stiffness (N/mm)	660 ± 92	516 ± 82^††^	609 ± 103**	667 ± 82**	728 ± 93**
Energy (N·mm)	83.0 ± 27.9	71.9 ± 24.4	79.8 ± 21.3	92.9 ± 26.6*	91.4 ± 21.3*

Values are shown as means ± SD

*Sham* sham surgery, *OVX* ovariectomy, *BMC* bone mineral content, *BMD* bone mineral density, *Cv/Av* cortical bone ratio, *Ct.Th* cortical bone thickness, *Ex.L* external length, *In.L* internal length, *Vv/Cv* cortical void volume

* *P* < 0.05 and ** *P* < 0.01: teriparatide versus OVX (Williams’ test for bone density and mechanical properties; Dunnett’s test for micro-CT); ^†^ *P* < 0.05 and ^††^ *P* < 0.01: OVX versus sham (*t* test)

### Mechanical Strength and the Relationships Between BMD and Bone Structure

Teriparatide significantly and dose-dependently increased the maximum load, stiffness, and breaking energy of the vertebra at doses ≥5.6 μg/kg compared with the OVX group (Table 1). Teriparatide also dose-dependently increased the mechanical strength of the femoral neck at doses ≥5.6 μg/kg compared with the OVX group (Table 2). Three-point bending tests of the femoral shaft revealed that teriparatide dose-dependently increased the ultimate load at doses ≥1.1 μg/kg. Teriparatide also increased stiffness and breaking energy of the femoral shaft at doses ≥1.1 and ≥5.6 μg/kg, respectively (Table 3).

**Fig. 4:**
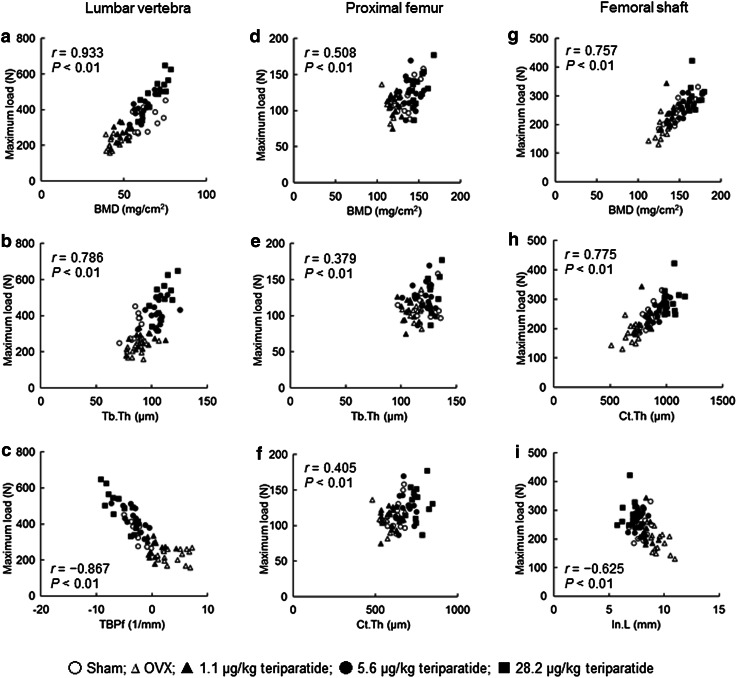
Correlations between the maximum load and bone density or structure parameters in the lumbar vertebra, proximal femur, and femoral shaft. *r* Spearman correlation coefficient, *P* P value

The relationships between mechanical strength (maximum load) and BMD or structural parameters of the lumbar vertebra, femoral neck, and femoral shaft are shown in Fig. 4 for all rats combined. The maximum load was significantly correlated with the BMD of the lumbar vertebra (*r* = 0.933; Fig. 4a), proximal femur (*r* = 0.508; Fig. 4d), and femoral shaft (*r* = 0.757; Fig. 4g). The maximum load of the lumbar vertebra was positively correlated with Tb.Th (*r* = 0.786) and was negatively correlated with TBPf (*r* = −0.867) (Fig. 4b, c). The maximum load of the proximal femur was positively correlated with Tb.Th (*r* = 0.379) and Ct.Th (*r* = 0.405) in the inter-trochanter region (Fig. 4e, f). The maximum load of the femoral shaft was positively correlated with Ct.Th (*r* = 0.775) and was negatively correlated with In.L (*r* = −0.625) (Fig. 4h, i).

### Stress Distribution and Fracture Load in the Proximal Femur

**Fig. 5:**
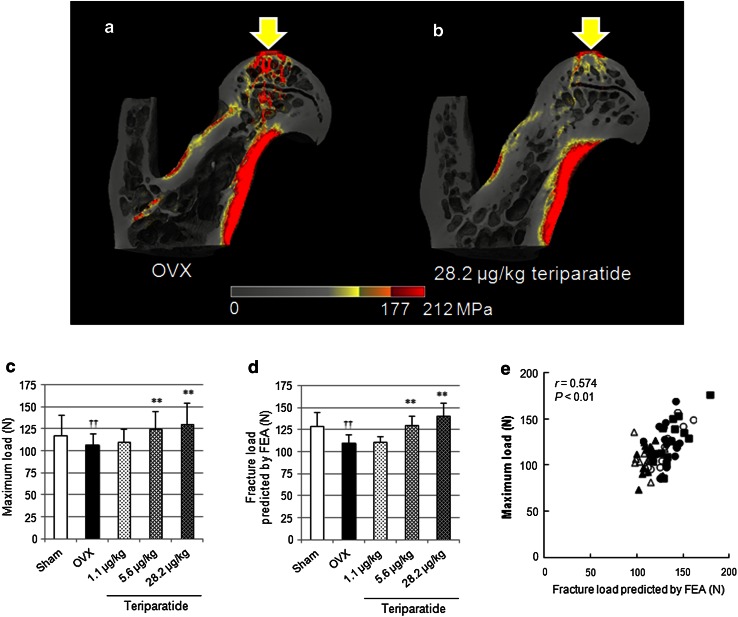
**a**, **b** Von Mises stress distribution with an axial compressive load on the *top* of the femoral head in proximal femoral models of the OVX (**a**) and 28.2 μg/kg teriparatide (**b**) groups. **c** Mechanical strength and **d** fracture load predicted by FEA. **P < 0.01: teriparatide versus OVX (Dunnett’s test); ^††^ *P* < 0.01: OVX versus sham (*t* test). **e** Correlation between the maximum load determined by mechanical testing and fracture load predicted by FEA

The distributions of von Mises stress in the proximal femoral models of the OVX group and high-dose teriparatide group are shown in Fig. 5a and b, respectively. In the OVX model, the high-stress voxels (>177 MPa; red) are widely distributed in the cortical bone of the shaft, in the trabecular bone of the femoral head, the neck, and the inter-trochanteric region. By contrast, high-stress voxels are only located in some cortical bone of the shaft in the high-dose teriparatide model.

The fracture load predicted by FEA was 15 % lower in the OVX group than in the sham group (110 vs. 129 N), which indicates that the deteriorations in structure and volumetric BMD in the OVX group attenuated bone strength. The fracture load was 18 and 29 % higher in the 5.6 and 28.2 μg/kg teriparatide groups, respectively (130 and 141 N, respectively; *P* < 0.01), but was only 1 % higher in the 1.1 μg/kg group (111 N). The fracture load determined by FEA (Fig. 5d) was positively correlated with the maximum load (Fig. 5c) determined by mechanical testing (*r* = 0.574; Fig. 5e).

## Discussion

There were four main results of this study with respect to the effects of three-times-weekly administration of teriparatide in OVX rats. (1) BMD increased dose-dependently in vertebral and peripheral bone sites (lumbar, tibia, and femur). (2) Serum osteocalcin increased without an increase in serum CTX. (3) The microstructure of trabecular and the cortical bone improved in dose-dependent manners. Tb.N and Tb.Th increased in the lumbar vertebral body and the proximal femur. Ct.Th increased toward the endocortical side of the proximal femur and femoral shaft. (4) The mechanical strength of the vertebral body, proximal femur, and femoral shaft increased in a dose-dependent manner, and the increases were correlated with BMD and bone microstructural properties.

In the present study, OVX rats were administered 1.1, 5.6, or 28.2 µg/kg teriparatide three-times-weekly. The 5.6 µg/kg dose in OVX rats was approximately twice that of a clinical dose of 56.5 µg/kg, administered once-weekly, in postmenopausal women based on a comparison of the areas under the concentration–time curves (data not shown). The dosing schedule was selected because the activation frequency of trabecular bone was reported to be ≥3 times greater in OVX rats [20–22] than in postmenopausal osteoporosis patients [23–26].

Earlier studies investigating the long-term daily administration of teriparatide in OVX rats [27, 28] did not report its effects on bone metabolism markers. In a prior study, daily administration of hPTH_1–84_ at a dose of 30 µg/kg (22 nmol/kg/week) for 12 months led to a 40 % increase in osteocalcin, a bone formation marker, relative to the control group [29]. In our study, three-times-weekly administration of teriparatide at 28.2 µg/kg (21 nmol/kg/week) elicited a similar increase in osteocalcin. However, in the previous study, daily hPTH_1–84_ increased urine deoxypyridinoline [29], a marker of bone resorption, whereas we observed no increase in serum CTX following three-times-weekly administration of teriparatide. These results suggest that daily administration of hPTH_1–84_ increases bone formation and bone resorption, with enhanced bone turnover favoring bone formation, whereas three-times-weekly administration of teriparatide may enhance bone formation with no accompanying increase in bone resorption.

In an earlier study in which teriparatide was administered once-daily for 12 months, the maximum load was correlated with BMC [28]. Daily administration of teriparatide or hPTH_1–84_ caused greater increases in BMC than BMD of the femur in OVX rats, resulting in an increase in the cross-sectional area of the femur. These changes were probably due to greater stimulation of periosteal and endocortical bone formation. The mechanical strength increased in relation to the increases in cross-sectional area and bone mass, compensating for the decrease in mineralization caused by enhanced bone turnover. In this study, we found that three-times-weekly administration of teriparatide increased the maximum load of the lumbar vertebra and femoral shaft, and that the maximum load was strongly correlated with BMD at both sites (*r* = 0.933 and *r* = 0.757, respectively). Regarding the cortical bone of the femoral shaft, although the external length was not affected by three-times-weekly administration of teriparatide, the maximum load was positively correlated with Ct.Th (*r* = 0.775) and negatively correlated with In.L (*r* = −0.625). These results suggest that three-times-weekly administration of teriparatide increases cortical thickness and bone density by promoting bone formation on the endocortical surface without promoting bone turnover, thereby increasing bone strength. In clinical settings, once-weekly administration of teriparatide increased cortical bone thickness without affecting the outer diameter of the femoral cortical bone [30], similar to our results.

Regarding trabecular bone of the lumbar vertebra, we found that three-times-weekly administration of teriparatide increased the mechanical strength, and these changes were positively correlated with increased Tb.Th and Tb.N. Teriparatide also improved trabecular structural features, such as TBPf. It was previously reported that daily administration of teriparatide increased Tb.Th and Tb.N [28], similar to the effects of three-times-weekly administration in our study. In clinical settings, daily and once-weekly administration of teriparatide significantly reduced the incidence of vertebral fracture, which might be due to positive effects of teriparatide on trabecular bone that are independent of its dosing frequency.

Unexpectedly, the correlations between maximum load and BMD (*r* = 0.508) and bone structure parameters of trabecular bone (Tb.Th: *r* = 0.379) and cortical bone (Ct.Th: *r* = 0.405) in the proximal femur were weaker than the correlations between similar parameters in the lumbar vertebra or femoral shaft. However, we found a positive correlation between the maximum load and the fracture load predicted by FEA based on micro-CT of the whole proximal femur (*r* = 0.574). These findings suggest that changes in the microstructure and mineralization of the entire proximal femur, not just changes in a single region, contribute to the increase in bone strength. The effects of teriparatide on bone formation are influenced by mechanical stress [31], and new bone formation preferentially occurs at sites with a high stress distribution [32]. Taken together, these results imply that three-times-weekly administration of teriparatide increases BMD in the proximal femur, and that these changes are accompanied by three-dimensional changes that contribute to increased mechanical strength.

Some limitations of this study need to be mentioned. We were unable to evaluate intracortical remodeling activity or intracortical porosity because rats have few Haversian systems. Additionally, the total doses of teriparatide used in a previous study (8 and 40 µg/kg, once-daily) were relatively high compared with the doses used in this study or in the clinical setting (20 µg, once-daily), and the difference in the effects on bones by daily and three-times weekly treatment may be partly affected by the treatment dose. Finally, we did not include control groups treated with teriparatide either once-daily or once-weekly to avoid confusion; instead, the comparisons with once-daily PTH were based on the results of similarly designed studies (i.e., multiple doses for 12 months in OVX rats). Further studies are needed to examine the functional impact of different administration frequencies of teriparatide, and this report provides essential information to support such studies.

In conclusion, the results of this study showed that three-times-weekly subcutaneous administration of teriparatide for 12 months increased bone formation without increasing bone resorption, with a modest increase in bone turnover in OVX rats. This regimen increased the mechanical loading strength of fracture-prone sites by improving the architecture of trabecular and cortical bone. Taken together, we believe that these results provide insights into the clinical effects of a lower frequent administration of teriparatide in humans, particularly in terms of increase in bone strength and reduced fracture risk.

## Electronic Supplementary Material

Supplementary material 1 (DOCX 24 kb)
