# Supplementary material for: Three-Times-Weekly Administration of Teriparatide Improves Vertebral and Peripheral Bone Density, Microarchitecture, and Mechanical Properties Without Accelerating Bone Resorption in Ovariectomized Rats
Source: Calcif Tissue Int. 2015 Apr 25;97(2):156–68. doi: 10.1007/s00223-015-9998-0 (PMC4491365; doi:10.1007/s00223-015-9998-0)
Supplement: Supplementary file 1 — Supplementary material 1 (DOCX 24 kb) [file 223_2015_9998_MOESM1_ESM.docx]

**Electronic Supplementary Materials**

**Three-times-weekly Administration of Teriparatide Improves Vertebral and Peripheral Bone Density, Microarchitecture, and Mechanical Properties Without Accelerating Bone Resorption in Ovariectomized Rats**

Ryoko Takao-Kawabata · Yukihiro Isogai · Aya Shimomura · Yukari Shimazu · Emika Sugimoto · Osamu Nakazono · Ichiro Ikegaki · Hiroshi Kuriyama · Shinya Tanaka · Hiromi Oda · Toshinori Ishizuya

**Contents**

Serum Biochemistry

Measurement of Bone Mineral Density (BMD)

Successful Establishment of Osteoporosis by Ovariectomy (OVX)

References

**Serum Biochemistry**

Osteocalcin was assayed as a bone formation marker using an enzyme-linked immunosorbent assay (ELISA) (Osteocalcin, rat ELISA System; GE Healthcare Bio-Science, Piscataway, NJ, USA). Type I collagen C-telopeptide was analyzed as a bone resorption marker using RatLaps ELISA (Nordic Bioscience Diagnostics, Herlev, Denmark). Calcium was measured using the methyl xylenol blue method (Calcium E-HA test Wako, Wako Pure Chemical Industries, Osaka, Japan). Inorganic phosphorous was assayed using the purine-nucleoside phosphorylase–xanthine dehydrogenase method (L-type Wako inorganic phosphorous, Wako Pure Chemical Industries). All assays were performed according to the manufacturers’ instructions, and were routinely performed within 3 months of sample collection.

**Measurement of Bone Mineral Density (BMD)**

BMD of the tibia, vertebral cylinders, and right femur was determined using dual energy X-ray absorptiometry (DXA; DCS-600EX-3R, Aloka, Tokyo, Japan).

BMD of the tibia was measured under ketamine and xylazine anesthesia (approximately 6.7 mg/kg ketamine and 3.3 mg/kg xylazine, intramuscular), at the following times: before surgery (−3M), and at 0, 3, 6, and 12M during the treatment period. We measured BMD in the tibia because metaphysis of long bones illustrates the effects of ovariectomy and parathyroid hormone treatment [[1](#_ENREF_1)]. The rats were placed in the prone position on the DXA scanning table. The left tibia was scanned at a pitch of 1 mm and scan speed of 25 mm/min. BMD was calculated from the values for bone mineral content and bone area for three regions of the tibia (proximal, shaft, and distal).

Bone density of the isolated lumbar vertebral cylinders and the right femora were measured using DXA (DCS-600EX-3R; Aloka). The specimens were placed on the scanning table ventral side up, and scanned at a pitch of 1 mm and scan speed of 25 mm/min. BMD was calculated from the values for bone mineral content and bone area. The femur was divided into three regions for analysis (proximal, shaft, and distal).

**Successful Establishment of Osteoporosis by Ovariectomy (OVX)**

As expected, OVX induced osteoporosis based on the observed changes in BMD and results of micro-CT (in-text Tables 1–3; in-text Fig. 3). Micro-CT (in-text Fig. 3) showed that OVX led to deteriorations in the trabecular architecture of the lumbar vertebra (in-text Table 1) at baseline and at the end of the study. Similar changes were also observed in the trabecular and cortical architectures of the proximal femur (in-text Table 1) and femoral shaft (in-text Table 2). The changes in micro-CT variables indicate that OVX led to a change in trabecular bone to an abnormal rod-like structure with low connectivity. Although OVX did not elicit clear changes in cortical bone in the femoral neck between the OVX and Sham groups (in-text Table 2), it did cause deteriorations in the micro-CT variables related to the femoral intra-trochanter and intra-trochanter cortical bone compared with the Sham group (in-text Table 2). OVX also led to deficits in BMD and the architecture of the femoral shaft compared with the Sham group; however, the change in the BMD of the cortical bone in the femoral shaft was slower than in cancellous bone-rich areas, similar to the changes in the tibial shaft. These changes in BMD and architecture in the OVX group were also accompanied by reductions in loading strength (ultimate load), stiffness, breaking energy of the lumbar vertebra, and ultimate load of the femoral shaft (in-text Tables 1–3). Taken together, these results confirm that OVX successfully induced osteoporosis in these rats.

**References**

1. Kishi T, Hagino H, Kishimoto H, Nagashima H (1998) Bone responses at various skeletal sites to human parathyroid hormone in ovariectomized rats: effects of long-term administration, withdrawal, and readministration. Bone 22:515-522
